# Outcome Analysis of Intramedullary Nailing Augmented with Poller Screws for Treating Difficult Reduction Fractures of Femur and Tibia: a Retrospective Cohort Study

**DOI:** 10.1155/2021/6615776

**Published:** 2021-04-02

**Authors:** Junfei Guo, Junpu Zha, Jun Di, Yingchao Yin, Zhiyong Hou, Yingze Zhang

**Affiliations:** ^1^Department of Orthopedic Surgery, Third Hospital of Hebei Medical University, Shijiazhuang, Hebei 050051, China; ^2^Orthopaedic Research Institute of Hebei Province, Shijiazhuang, Hebei 050051, China; ^3^Key Laboratory of Biomechanics of Hebei Province, Shijiazhuang, Hebei 050051, China; ^4^NHC Key Laboratory of Intelligent Orthopaedic Equipment, The Third Hospital of Hebei Medical University, Shijiazhuang, Hebei, China; ^5^Chinese Academy of Engineering, Beijing 100088, China

## Abstract

**Purpose:**

Poller screws may serve as an adjunctive reduction tool and aid fracture reduction while augmented with intramedullary (IM) nailing for treating diaphyseal or metaphyseal fractures of the femur and tibia. However, there is no consistent conclusion about whether the method of using IM nailing augmented with poller screws is more advantageous than using IM nailing alone.

**Methods:**

A total of 96 patients who received IM nailing with or without supportive poller screw for treating long-bone fractures in lower limbs and who experienced difficulties in performing reduction or IM insertion during the surgical process were included in this retrospective cohort study (33 patients with poller screws in group A versus 63 patients without poller screws in group B). Patient demographics including age, gender, and body mass index; injury-related data including fracture location, classification, and injury mechanism; operation-related data including American Society of Anesthesiologists, duration of operation, poller screw time, method of anesthesia, and volume of intraoperative hemorrhage; outcomes including fracture healing time; and incidence of outcomes of nonunion, malunion, infection, and secondary surgical procedures were evaluated.

**Results:**

Fracture healing time of patients in group A was significantly shorter than that of group B (18.3 ± 4.8 weeks versus 24.3 ± 3.0 weeks, *p* = 0.023). Union rate was higher (100.0% versus 87.3%, *p* = 0.048), and malunion rate and secondary surgical procedure rate were lower (both are 3.0% versus 19.0%, *p* = 0.031) in group A than that of group B.

**Conclusion:**

Poller screw augmentation of IM nailing is a favourable option to shorten fracture healing time and to reduce complication rates in terms of nonunion, malunion, and secondary surgical procedure in the treatment of both diaphyseal/metaphyseal fractures of the femur or tibia while compare with those treated by IM nailing alone.

## 1. Introduction

Intramedullary (IM) nail fixation has become the preferred choice of operative management of long-bone fractures of the lower limb due to its numerous advantages such as minimally invasive, limited soft tissue dissection, short hospital stay, and early weight bearing, thus making it superior compared to plates and screws [1–3]. In recent years, indications of IM nailing have been extended to metaphyseal fractures [4]. However, one of the major limitations with the “standard” practice of IM nailing in the treatment of metaphyseal fractures is different sizes between the nail diameter and the metaphyseal diameter, making the bone-implant contact small, which decreases biomechanical stability and frequently result in malalignment and nonunions and requires reoperations [4–12].

The term “blocking screw” was firstly introduced by Donald and Seligson in 1983 [13]. To prevent axial deformities in proximal or distal third tibia fractures during IM nailing, “poller screw” was firstly described by Krettek in 1999 [14], which was similar to the metal devices designed to block or guide traffic. The screw, as an effective and valuable intraoperative tool, was thought to work by decreasing the width of the medullary canal in metaphysis and providing a tight mechanical fit for the IM nail, and then increasing the stability of the bone-implant construct. Due to the advantages that they do not require special hardware and no need for excessive soft tissue dissection, a number of previous studies have shown that IM nailing augmented with poller screws may help in reducing deformity in axis, controlling angular deformity, and effectively aiding fracture reduction by directing the IM nail during insertion [3, 5, 15–19].

To the best of our knowledge, the placement of poller screw may not be successful or achieved for one time due to the different technical experience among surgeons. Additional drilling attempts also prolong the operation time as well as increasing the possibility of complications. For this reason, anecdotally, poller screws as a technical challenge are used by experienced surgeons in large trauma centers rather than trainee surgeons [20]. As a result, numerous studies reported nonunions [10, 21], coronal malalignment [10, 22, 23], sagittal malalignment [24], complication of superficial [21]/deep infection [14, 22, 23], and requirement of secondary surgical procedures [10] in patients treated with poller screws.

Overall, there is no consistent conclusion about whether the method of using IM nailing augmented with poller screws is more advantageous than using IM nailing alone, as there is a paucity of data comparing their efficiency and efficacy. Therefore, we conducted this retrospective study that compared the outcomes between IM nailing augmented with poller screws and IM nailing alone for treating diaphyseal or metaphyseal fractures of femur and tibia.

## 2. Materials and Methods

### 2.1. Patients and Groups

The study population consisted of patients with diaphyseal or metaphyseal fractures of the femur and tibia and, all patients experienced difficulties in performing reduction or IM insertion during the surgical process at a single Level I trauma center in China between January 2014 and May 2018. We included patients with acute fractures, who had an admission delay less than 48 hours, who received IM nail treatment with or without poller screws, and patients for whom there were sufficient available radiographs until union. Patients who had multiple fractures or injuries, pathologic or open fractures, nondisplaced fractures, adolescent patients, deep intramedullary infection cases, those who were treated conservatively, and patients without sufficient radiographic follow-up were excluded.

Patients were retrospectively assigned to two groups according to their treatment: group A of patients using IM nailing augmented with poller screws and group B of patients received IM nail treatment alone. This study was approved by the institutional review board of the third Hospital of Hebei Medical University in compliance with the Helsinki, and declaration and consent were waived for its retrospective nature.

### 2.2. Surgical Procedure and Technical Description

X-rays of the injured leg in positive and lateral views were taken, and fractures were classified according to the AO/OTA classification. All fractures were also analyzed using preoperative CT imaging to determine the configuration of fracture patterns. In accordance with the fracture pattern, metaphyseal fractures were stabilized with appropriate locked intramedullary nails on a standard radio lucent table. Tourniquet was used if necessary. Through the entry point, guide wire was passed under image control. Closed reduction was then done in all cases.

**Figure 1:**
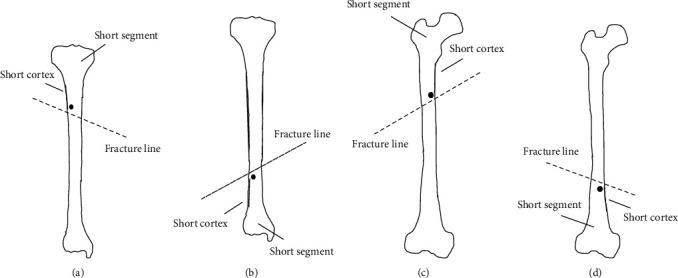
Illustrations showing the best location placement of the poller screw on the coronal plane. The Poller nail should be placed perpendicular to the deformity plane on the short fracture segment and short cortex close to the intramedullary nail. The short fracture segment and short cortex in proximal tibia fracture (a), distal tibia fracture (b), proximal femur fracture (c), and distal femur fracture (d) are shown.

Poller screws were placed perpendicular to the deformity plane on the short bone segment and the short cortex side close to the intramedullary nail (Figure 1), which we called the “short-short principle” in our poller screw placing technique. Under careful image fluoroscopy, the poller screw should be accurately placed as close to the fracture line as possible while avoiding any comminution with this principle before reduction with final nail insertion since drill bit may damage the nail while drilling with the nail in situ. If the intramedullary nail has tendency to migrate in the coronal plane, a Kirschner wire was used as a drill drilling in anteroposterior place until it has perforated the opposite cortex. Then, a poller screw is substituted for the Kirschner wire. Finally, the right path for the nail can be achieved, and the reamer will ream toward the correct direction. After achieving the alignment using blocking screws, the distal and proximal locking was then done. After that, the alignment was confirmed in both coronal and sagittal planes with image fluoroscopy.

**Figure 2:**
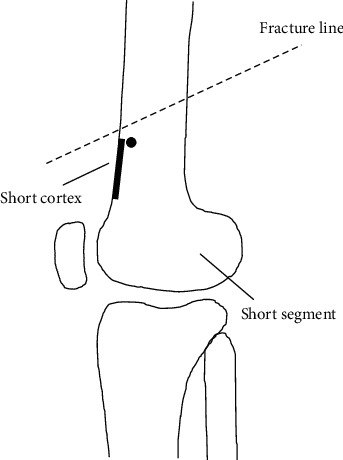
Illustrations showing the best location placement of the poller screw on the sagittal plane. The short fracture segment and short cortex in distal femur fracture with sagittal displacements are shown in the lateral view.

For malaligned fractures, first, place a poller screw with IM nail temporarily removed and then reinsert the nail. In addition, after the nail has been placed, the poller screw should not be adjusted iteratively because correcting the deformity then becomes much more difficult. Our experience showed the malunion is more prone to occur in the coronal plane than the sagittal plane; however, our method is also suitable for sagittal displacements in the lateral view (Figure 2). With spiral fractures, a single poller screw may be insufficient to provide enough stability in all planes. To avoid failure, we suggest one poller screw in sagittal and another in coronal plane depending on fracture pattern to fully control alignment.

After operation, we recommended partial weight bearing that started in the second postoperative week. Patients were then followed up with periodic reexaminations, and timing of partial-to-full weight bearing was decided according to the clinical and radiological evidence of union.

### 2.3. Data Extraction

The collected data of interest included demographics including age, gender, and body mass index (BMI); injury-related data including fracture location (proximal and distal fracture of the femur and tibia), classification (AO/OTA), and injury mechanism; operation-related data including American Society of Anesthesiologists (ASA, six grade), duration of operation, poller screw time, method of anesthesia (general anesthesia, regional anesthesia), and volume of intraoperative hemorrhage; outcomes including fracture healing time (radiological union), and incidence of outcomes of nonunion, malunion (Angulation > 5° on any plane [24], rotational deformity > 15° [10], or shortening > 2 cm [25]), infection (superficial/deep infection), and secondary surgical procedures.

The BMI was recorded as normal with BMI < 24 kg/m^2^, overweight with 24 ≤ BMI < 28 kg/m^2^, and obesity with BMI ≥ 28 kg/m^2^. Union was defined as the ability to bear full weight without pain, with the callus bridging in 3 of 4 cortices on radiographs while nonunion was defined as the absence of progressive fracture healing for three consecutive months. Infections were classified as superficial and deep infections. Superficial infections were defined as wound infections that resolved with antibiotic treatment without surgical intervention, while deep infections were defined as infections requiring surgical debridement or diagnosis of osteomyelitis. The mechanism of injury was classified as low-energy fracture caused by fall from standing height or bicycle injury, and high-energy fracture from high height or motor vehicle injury.

### 2.4. Statistical Analysis

The continuous variables were evaluated for normality by using the Shapiro-Wilk test. Data satisfying normality are presented as the mean ± standard deviation. The tests for significant differences between normally distributed data samples were performed using Student's *t* test. Categorical data are presented as absolute numbers (percentages), and the Chi-square or Fisher's exact tests were used to compare patient number distributions between the groups. All data analyses were performed using the IBM SPSS Statistics for Windows, version 26.0 (IBM, Armonk, NY, USA). The level of significance was set at *p* < 0.05.

## 3. Results

From January 2015 to May 2018, a total of 96 patients met the inclusion criteria and were evaluated. 33 patients who underwent IM nailing augmented with poller screws were classified as group A, and 63 patients who underwent IM nail treatment alone were classified as group B. There were 68 male and 28 female patients. The mean (range) follow-up time was 31.8 (28-46) months, and the mean (range) age of included patients was 48.0 (20-94) years. The distribution patient demographic characteristics and injury-related data are displayed in Table 1. There were no statistically significant differences between the two groups for any parameter.

Table 2 showed no statistical difference in the mean duration of operation between the two groups (139.1 ± 31.0 min for group A vs. 137.2 ± 29.4 min for group B), although placing poller screws took a mean of 26.2 ± 5.1 minutes extra operation time in group A. There were also no significant differences in other operation-related data including ASA grade, method of anesthesia, and volume of intraoperative hemorrhage (*p* > 0.05). The fracture healing time of patients in group B was significantly longer than that of group A (24.3 ± 3.0 weeks in group B vs. 18.3 ± 4.8 weeks in group A, *p* = 0.023).

Eight patients (all in group B) had nonunion, among which, one was hypertrophic nonunion, then underwent additional poller screw insertion, and finally obtained union. Another four had atrophic nonunion and resolved by bone grafting and exchange nailing, and union was obtained 15 to 18 weeks later. The other three nonunion patients had oligotrophic nonunion, with distal interlocking screw breakage four to seven weeks after surgery. After reinserting the distal interlocking screw and the additional poller screws, union was achieved 16-19 weeks later. The union rate was significantly higher (100.0% vs. 87.3%, *p* = 0.048) in the poller screw group (group A) than that in IM nail alone (group B). There was one patient in group A and twelve patients in group B of malunion according to our definition, which had statistically significant differences (*p* = 0.031). Of the one patient in group A and twelve patients in group B, both with malunion in group B, all had deformities due to initial insufficient reduction. However, because these patients had no clinical discomfort in their daily lives, additional surgical procedures are unnecessary.

In terms of infection, no relevant differences between groups are to be expected (*p* > 0.05). A total of eight patients (two in group A and six in group B) had superficial infection while two patients (all in group B) had deep infection. Of the patients with superficial infection, all were wound infections at sutures or entry points that healed after antibiotic treatment and incisional drainage or debridement and without sequelae. During the study period, secondary procedure was required in only one case in group A and twelve cases in group B to achieve union (*p* = 0.031). Among the twelve cases in group B, eight patients were nonunion, another four were superficial or deep infection (each with two cases). The reason for secondary surgical procedure of the patient in group A was superficial infection. The detailed characteristics of operation-related data and outcomes are presented in Table 2.

## 4. Discussion

In recent years, intramedullary nailing has become the standard treatment for diaphyseal tibial and femoral fractures. However, it is associated with an increased risk of nonunion or malalignment [6–8, 26, 27]. Previous researches have shown that fractures of the distal third of tibia treated with IM nailing frequently result in varus/valgus, torsional deformities and nonunions [5–8], which is the same with the femur [10–12]. The incidence in malalignment is reported to be as high as 14%-58% for tibia fractures [15, 28] and 10%-30% for femur fractures [11]. And IM nailing after tibial/femur shaft fractures still demonstrates nonunion rates of 5% to 25% [29–31] and 0.5% to 12.5% [32–37], respectively.

The most possible factor determining the occurrence of these complications might be instability caused by different sizes between the nail diameter and the metaphyseal diameter. Mugundhan et al. analyzed the mismatch between the diameters of the medullary canal at the level of isthmus (as maximum nail size) and at the fracture site in 20 cases, whose results showed there was a significant mismatch that leads to the metaphysis with no nail-cortex contact [22]. Additionally, malalignment as another important reason is associated with the muscular force imbalance [10], which can occur even if the intramedullary nail is interlocked, though the interlocking screw has been proved to be good at controlling the limb length and rotation of the fragments. In the absence of metaphyseal cortex contact, the intramedullary nail may translate along the interlocking screw, especially in the cases with fractures more than one plane [5, 38]. In a research of 386 fractures of tibia treated by intramedullary nailing, Ahlers and von Issendorff [27] found that one-quarter to one-third had varus/valgus deformities greater than 4 degrees. Similarly, Van et al. [26] reported a 15-year follow-up of 88 patients with fractures of the lower leg, 49% had healed with malalignment of at least 5 degrees. They also found degenerative changes that more arthritis occurred in the knee and ankle adjacent to fracture than in comparable joints of the uninjured leg. Others also suggested that even if adding a sagittal interlocking nail cannot prevent the angular displacement in the coronal plane, and they called it “risk of toggling around the nail” [15, 39].

By aligning nails in long-bone fractures and creating a narrow rigid canal to centralize the nail, the poller screw has been purported to expand the indication for intramedullary nailing and reduce rates of nonunion and malunion seen with IM nailing of metaphyseal fractures [20]. During reduction, poller screws are placed adjacent to the nail and perpendicular to the screw holes usually in an anteroposterior direction [22] to provide a tight tunnel by decreasing the width of medullary cavity and to guide the nail in the center with the wrong nail path blocked. It has also been described increasing the mechanical stiffness of the bone-implant construct by using as a supplement to obtain three-point fixation, together with the other two points of fixation: first, the entrance point of the nail (when in distal third fracture) or the anchorage of its tip (when in proximal third fracture) and, second, the cortical wall through the isthmus in the long segment [16, 40].

Despite poller screw biomechanics having been clearly described, there has been no agreement on the outcomes of IM nailing augmented with poller screws. Various studies [14, 16, 19, 21, 41] have showed the efficacy of poller screws combined with IM nail in treating femoral and tibial fractures. They were proved to be associated with balance of soft tissue tension, decreasing the fracture displacement or nonunion, as well as reducing the risk of implant failure [19, 21, 25, 41]. However, in a more recent retrospective study on 116 femur fractures [38], Van Dyke et al. found there was no statistically significant difference in the time to union, union rate, and the overall alignment between IM groups with or without the use of poller screws. However, the author also revealed in their study that poller screws were used more often in difficult cases, which may represent selection bias to obscure the results. In this regard, we performed the current study addressing this issue specifically with all patients selected in the cohort experienced difficulties in performing reduction or IM insertion during the surgical process.

Our results showed lower complication rates of IM nailing augmented with poller screws than IM nailing alone in terms of nonunion (0.0%), malunion (3.0%), and secondary surgical procedures (3.0%) and shorter fracture healing time (with all *p* values < 0.05.). And our data also revealed that the operation-related data including ASA grade, duration of operation, volume of intraoperative hemorrhage, and complication rate of infection (both superficial and deep) in IM nailing augmented with poller screws group were statistically noninferior to the group of IM nailing alone (with all *p* values > 0.05.). All of our results compared favourably to the complication rates reported in previous studies of the treatment of metaphyseal fractures with IM nailing alone, where previous researches indicated the ranges of nonunion rates of 5.5% 12, malunion rates of 8.2%, 16.2%, infection rates of 4.3%, and secondary surgical procedure rates of 16.4% [28, 42, 43]. This is consistent with the previous study [17] which indicated poller screws can be used for alignment control in osteotomy of metaphyseal bone. In addition, Krettek et al. [41] created bone-implant construction in the fresh cadaveric tibia and demonstrated the application of poller screws decreasing the deformation by 25% in proximal fractures and 57% in distal fractures.

Our complication rates were also favourable or comparable when compared to a systematic review on the outcomes of IM nailing augmented with poller screws in treating long-bone fracture, with the average rates of nonunion of 4.0%, malunion rates of 5.0%, superficial infection rates of 6.0%, deep infection rates of 5.0%, and secondary surgical procedure rates of 8.0% [20]. It might benefit from the practical method used in this study that ensures the proper placement of the poller screw for maximizing the benefits. Furthermore, strict patient selection criteria add strength to the study result.

To the best of our knowledge, placing the poller screws away from the desired location can lead to repeated operations, risk of screw bending, and the damage of reamers and nails [3, 18]. Therefore, how to determine the accurate position of Poller screw is of significant importance. Multiple techniques have been reported for determining the placement of poller screw, and the most frequent described placement is on the concave side of the deformity, closed to the fracture site and in the short bone segment [14, 16–19, 21, 22, 25, 41]. Stedtfeld et al. [16] and Shahulhameed et al. [18] suggested placing the poller screws in different fracture sites involving the proximal and distal metaphysis of long bones. Gao et al. [25] and Yoon et al. [3] described placing it according to the potential translation direction of the short bone segment. Hannah et al. [44] described a seven-step placement method to correct the reduction by placing the poller screw in acute angles formed in the oblique fracture pattern. Muthusamy et al. [39] introduced the “reverse rule of thumb” principle as a quick reference to determine the ideal locations and number of blocking screws. More recently, Goldzak et al. [5] proposed a novel distal tibial fracture classification, according to which the poller screw location was selected. In all these extant methods, the technique cannot be applied to all types of tibia/femoral fractures and possibly regard to the orientation of fracture line, which should all be taken into account for the poller screw placement. It is too complicated and confusing to understand for surgeons, especially for young trainees, while in clinical practice, that creates a pause in thinking carefully about deforming forces.

Based on this, we describe a simple, practical, and easy-to-remember method that permits achieving the best placement of the poller screw by using the “short-short principle” without pause for thinking. The principle helps ensuring the proper placement of the poller screws to obtain maximum benefit, and it can be applied to any proximal or distal segment of long-bone fracture with antegrade or retrograde techniques, no matter in either coronal or sagittal plane. Only very few comminuted fractures and transverse fractures need to judge the potential orientation of the fracture displacement.

There are several limitations associated with our study. Firstly, the data was collected in a single center. Secondly, we only conducted X-ray examination rather than CT scan for malalignment due to the economic burden. Another limitation was that we could not do more stratified analyses due to fewer number of participants included or handle advanced analyses such as multiple regression. However, we proposed a novel simple, practical, and easy-to-remember method that permits achieving the best placement of the poller screw without pause for thinking. And the specific cohort of patients received surgeries with IM nailing augmented with poller the screws by the same method, which eliminated the effects of possible confounding variables. Another strength of this study was that the patients involved all experienced difficulties in performing reduction or IM insertion during the surgical process that avoids the selection bias.

## 5. Conclusion

In conclusion, the poller screws augmentation of IM nailing is a favourable option to shorten fracture healing time and to reduce complication rates in the treatment of both diaphyseal/metaphyseal fractures of the femur or tibia comparing with those treated by IM nailing alone, especially in difficult cases of performing reduction or IM insertion during the surgical process.

## Figures and Tables

**Table 1:** Comparisons of patient demographics and injury-related data between two groups^∗^.

Characteristics	Overall (*n* = 96)	Group A (*n* = 33)	Group B (*n* = 63)	*p* value
Demographic				
Age^#^, years	48.0 ± 18.6	43.9 ± 16.9	50.2 ± 19.3	0.115
Gender, no. (%)				
Male	68 (70.8%)	27 (81.8%)	41 (65.1%)	0.087
Female	28 (29.2%)	6 (18.2%)	22 (34.9%)	
BMI group, no. (%)				0.570
Normal (BMI<24 kg/m^2^)	38 (39.6%)	13 (39.4%)	25 (39.7%)	
Overweight (24 ≤ BMI < 28 kg/m^2^)	48 (50.0%)	18 (54.5%)	30 (47.6%)	
Obesity (BMI ≥ 28 kg/m^2^)	10 (10.4%)	2 (6.1%)	8 (12.75)	
Injury-related data				
Fracture location, no. (%)				0.223
Proximal femur	36 (37.5%)	8 (24.2%)	28 (44.4%)	
Distal femur	25 (26.0%)	9 (27.3%)	16 (25.4%)	
Proximal tibia	13 (13.5%)	6 (18.2%)	7 (11.1%)	
Distal tibia	22 (23.0%)	10 (30.3%)	12 (19.1%)	
Fracture classification, no. (%)				
Femur				0.436
A	40 (65.6%)	9 (52.9%)	31 (70.5%)	
B	18 (29.5%)	7 (41.2%)	11 (25.0%)	
C	3 (4.9%)	1 (5.9%)	2 (4.5%)	
Tibia				0.686
A	24 (68.6%)	11 (68.8%)	13 (68.4%)	
B	8 (22.9%)	3 (18.8%)	5 (26.3%)	
C	3 (8.5%)	2 (12.4%)	1 (5.3%)	
Injury mechanism, no. (%)				0.408
Low-energy fracture	55 (57.3%)	17 (51.5%)	38 (60.3%)	
High-energy fracture	41 (42.7%)	16 (48.5%)	25 (39.7%)	

∗The differences between the groups were not statistically significant for all parameters. ^#^The values are given as the mean and the standard deviation.

**Table 2:** Comparisons of operation-related data and outcomes between two groups.

Characteristics	Overall (*n* = 96)	Group A (*n* = 33)	Group B (*n* = 63)	*p* value
Operation-related data				
ASA grade, no. (%)				0.833
I	45 (46.9%)	15 (45.4%)	30 (47.6%)	
II	32 (33.3%)	12 (36.4%)	20 (31.7%)	
III	12 (12.5%)	3 (9.1%)	9 (14.3%)	
IV	7 (7.3%)	3 (9.1%)	4 (6.4%)	
Duration of operation^#^, min	137.9 ± 29.8	139.1 ± 31.0	137.2 ± 29.4	0.772
Poller screw time^#^, min	NA	26.2 ± 5.1	NA	
Method of anesthesia, no. (%)				0.978
General anesthesia	38 (39.6%)	13 (39.4%)	25 (39.7%)	
Regional anesthesia	58 (60.4%)	20 (60.6%)	38 (60.3%)	
Volume of intraoperative hemorrhage^#^, mL	389.3 ± 225.1	375.2 ± 243.1	396.8 ± 216.8	0.658
Outcomes				
Fracture healing time^#^, weeks	19.7 ± 3.8	18.3 ± 4.8	24.3 ± 3.0	0.023^∗^
Nonunion, no. (%)				0.048^∗^
Yes	8 (8.3%)	0 (0.0%)	8 (12.7%)	
No	88 (91.7%)	33 (100.0%)	55 (87.3%)	
Malunion, no. (%)				0.031^∗^
Yes	13 (13.5%)	1 (3.0%)	12 (19.0%)	
No	83 (86.5%)	32 (97.0%)	51 (81.0%)	
Infection, no. (%)				
Superficial infection				0.711
Yes	8 (8.3%)	2 (6.1%)	6 (9.5%)	
No	88 (91.7%)	31 (93.9%)	57 (90.5%)	
Deep infection				0.544
Yes	2 (2.1%)	0 (0.0%)	2 (3.2%)	
No	94 (97.9%)	33 (100.0%)	61 (96.8%)	
Secondary surgical procedures				0.031^∗^
Yes	13 (13.5%)	1 (3.0%)	12 (19.0%)	
No	83 (86.5%)	32 (97.0%)	51 (81.0%)	

^#^The values are given as the mean and the standard deviation. ∗*p* < 0.05, statistical significance.

## Data Availability

The data presented in this study are available on request from the corresponding author.
